# Association between dietary caffeine intake and severe headache or migraine in US adults

**DOI:** 10.1038/s41598-023-36325-8

**Published:** 2023-06-23

**Authors:** Lu Zhang, Jiahui Yin, Jinling Li, Haiyang Sun, Yuanxiang Liu, Jiguo Yang

**Affiliations:** 1 The First Clinical College, Shandong University of Traditional Chinese Medicine, Jinan, China; 2 College of Traditional Chinese Medicine, Shandong University of Traditional Chinese Medicine, Jinan, China; 3 College of Acupuncture and Massage, Shandong University of Traditional Chinese Medicine, Jinan, China; 4 The Second Clinical Medical College, Guangzhou University of Chinese Medicine, Guangzhou, China; 5 Department of Neurology, Affiliated Hospital of Shandong University of Traditional Chinese Medicine, Jinan, China

**Keywords:** Nutrition, Risk factors

## Abstract

The relationship between current dietary caffeine intake and severe headache or migraine is controversial. Therefore, we investigated the association between dietary caffeine intake and severe headaches or migraines among American adults. This cross-sectional study included 8993 adults (aged ≥ 20 years) with a dietary caffeine intake from the National Health and Nutrition Examination Surveys of America from 1999 to 2004. Covariates, including age, race/ethnicity, body mass index, poverty-income ratio, educational level, marital status, hypertension, cancer, energy intake, protein intake, calcium intake, magnesium intake, iron intake, sodium intake, alcohol status, smoking status, and triglycerides, were adjusted in multivariate logistic regression models. In US adults, after adjusting for potential confounders, a 100 mg/day increase in dietary caffeine intake was associated with a 5% increase in the prevalence of severe headache or migraine (odds ratio [OR] 1.05, 95% confidence interval [CI] 1.02–1.07). Further, the prevalence of severe headache or migraine was 42% higher with caffeine intake of ≥ 400 mg/day than with caffeine intake of ≥ 0 to < 40 mg/day (OR 1.42, 95% CI 1.16–1.75). Conclusively, dietary caffeine intake is positively associated with severe headaches or migraines in US adults.

## Introduction

Severe headache or migraine is a common neurological disorder that can seriously affect people's daily lives and heavily burden individuals and society^1^. Globally, severe headache or migraine ranks second among the causes of years lived with disability, with the greatest age-standardized prevalence in 1990 and 2017^2^. It is three times more common in women than men, with a lifetime prevalence of 43% and 18%, respectively^3^. It remains a serious public health issue in the United States (US), with an age-adjusted prevalence of 15.9% across all adults in 2018^4^. The financial strain of migraine is enormous; approximately 40% of US adults with migraine were unemployed in 2018, and a similar percentage were classified as poor or “near poor”^5^. Therefore, effective preventive measures and modifiable risk factors for severe headaches or migraines should be investigated.

Recent studies have shown that genetics, sleep, and diet are contributing factors to headaches^6–8^. Caffeine is an important area of concern in diet, and it occurs naturally in various foods, such as coffee beans, tea, kola nuts, mate leaves, and cocoa nuts^9^. Caffeine is an antagonist of adenosine, inhibiting the release of excitatory neurotransmitters, resulting in decreased cortical excitability^10,11^. Additionally, caffeine has psychostimulant effects via the modulation of dopaminergic neurons, and dopamine plays a role in the pathogenesis of migraine^12,13^. Headache attacks are related to changes in cerebral blood flow, and caffeine intake or withdrawal can change the speed of cerebral blood flow and aggravate headaches^14,15^, since it significantly affects the central nervous system^16,17^.

Previous studies have reported the wide use of caffeine for patients with headaches, either alone or in combination with other treatments^18^. The American Headache Society recommends over-the-counter (OTC) nonsteroidal anti-inflammatory drugs (NSAIDs) and combinations such as acetaminophen, aspirin, and caffeine as Level A recommendations for reducing migraine and other symptoms^19^. Derry et al. reported in a randomized double-blinded study that the addition of caffeine (≥ 100 mg) to standard doses of commonly used analgesics improved pain relief^20^; however, this finding varies among studies. Shirlow et al. conducted a study on Australians and reported that the proportion of participants with headaches increased significantly with average caffeine intake^21^. However, Boardman et al. found no clear relationship between caffeine intake and headache in a cross-sectional study in the United Kingdom (UK)^22^. In another study, Hoy et al. reported that caffeine preparations were effective and generally well tolerated in treating migraine and episodic tension-type headache (TTH) in adult patients enrolled in a randomized, multicenter, active-comparison study^23^. Most previous studies are based on surveys of small samples, and studies on adults are insufficient^24–27^. Therefore, it is necessary to further investigate the relationship between dietary caffeine and severe headaches or migraines and explore the underlying mechanisms. Ultimately, we used data from the National Health and Nutrition Examination Survey (NHANES) database to explore the association of dietary caffeine intake with severe headaches or migraine in adults.

## Methods

### Study design and participants

The data on study participants were obtained from the NHANES, a major program conducted by the Centers for Disease Control and Prevention (CDC) to assess the health and nutritional status of adults and children in the US^28^. The NHANES database contains demographic, dietary, examination, laboratory, and questionnaire data. Study protocols for NHANES were approved by the National Center for Health Statistics (NCHS) Ethics Review Board (Protocol #98-12, https://www.cdc.gov/nchs/nhanes/irba98.htm). All the participants signed the informed consent before participating in the study. This study was conducted in accordance with the Declaration of Helsinki (as revised in 2013). All information from the NHANES program is available and free to the public; therefore, the agreement of the medical ethics committee board was not necessary.

Participants in our study were screened according to the following inclusion criteria: (1) aged 20 years or above, (2) caffeine intake was obtained through at least one 24-h recall, and (3) information on whether they had severe headaches or migraines.

### Assessment of severe headache or migraine

Severe headache or migraine was assessed using a questionnaire that consisted of one question: “During the past 3 months, did you have severe headaches or migraines?” Participants who answered yes were considered to have severe headaches or migraine, and participants who answered no were considered not to have severe headaches or migraine.

### Assessment of dietary caffeine

Dietary caffeine intake was collected through two 24-h dietary recall interviews. The first 24-h dietary recall was conducted in person, and the second was conducted 3–10 days later via telephone. The in-person interview was conducted in a private room in the NHANES mobile examination centre using a computer-assisted dietary interview system, which an NHANES interviewer administered. The amount of caffeine consumed (mg/day) was estimated from all caffeine-containing foods and beverages, including coffee, tea, soda, and chocolate. The caffeine content of all foods consumed by participants was estimated using the United States Department of Agriculture’s Food and Nutrient Database. We obtained the daily caffeine intake based on the sum of the caffeine content of all foods consumed in a single 24-h dietary review. Detailed information on the assessment of caffeine intake can be found at: https://wwwn.cdc.gov/Nchs/Nhanes/1999-2000/DRXIFF.htm#DRXICAFF.

In this study, we extracted the mean caffeine intake between the first and second dietary recalls as participants’ dietary caffeine intake. For participants who only attended one 24-h dietary recall, caffeine intake was defined as the day's caffeine intake.

### Assessment of covariates

Covariates in this study, including age, triglycerides (TG), energy intake, protein intake, calcium intake, magnesium intake, iron intake, and sodium intake, were used as continuous variables. Categorical variables included education level (< high school, completed high school, > high school), race/ethnicity (non-Hispanic white, non-Hispanic black, or others), and marital status (married/living with a partner or widowed/divorced/separated/never married). Poverty-income ratio (PIR) was defined as the ratio of family income to poverty threshold (< 1 indicating an income below the poverty threshold and ≥ 1 indicating an income above the poverty threshold; the latter category was further divided into two groups: 1.00 to < 2.00, ≥ 2.00). Body mass index (BMI) was measured as weight (kg) divided by height (m) squared (< 25.0 kg/m^2^ indicating normal, 25.0 to < 30.0 kg/m^2^ indicating overweight, ≥ 30.0 kg/m^2^ indicating obese). Smoking status (never smoking, < 100 cigarettes; former smoker, not currently smoking but ≥ 100 cigarettes consumed previously; current smoker, ≥ 100 cigarettes and currently smoking every day or some days). Alcohol status was determined by whether the participant had at least 12 alcoholic drinks per year (yes or no). Cancer was judged by answering the following question: “Have you ever been told by a doctor or other health professional that you had cancer or a malignancy of any kind?” (yes or no). Hypertension (defined as systolic blood pressure ≥ 140 mmHg or diastolic blood pressure ≥ 90 mmHg) was determined by either three blood pressure measurements at different times, an existing diagnosis, or evidence of an existing antihypertensive medication regimen.

### Statistical analysis

The main concern was whether dietary caffeine intake was associated with severe headaches or migraine after adjusting for other factors that may influence severe headaches or migraines. Continuous variables are expressed as mean ± standard deviation, and categorical variables are expressed as percentages. The χ^2^ test was used to compare categorical variables between groups, a one-way analysis of variance was employed to compare normally distributed variables between groups, and the Kruskal–Wallis H test was utilized to compare variables with a skewed distribution between groups. Multivariable logistic regression analysis was performed to evaluate the independent association between dietary caffeine intake and severe headaches or migraine. Meet the methodological requirements. We used four levels of adjustment: Model 1 was adjusted for age and race/ethnicity; Model 2 was adjusted for the variables in Model 1 plus BMI, PIR, educational level, marital status, alcohol status, and smoking status; Model 3 was adjusted for the variables in Model 2 plus hypertension, cancer, and triglycerides; Model 4 was adjusted for the variables in Model 3 plus energy intake, protein intake, calcium intake, magnesium intake, iron intake, and sodium intake. Missing values were assigned as dummy variables and included in the regression equation; the missing values are presented in Table S1.

All analyses were performed using R (The R Foundation, Vienna, Austria) and Empower (X&Y Solutions, Boston, MA, USA). Statistical significance was defined as a two-sided *P* value of < 0.05.

## Results

### Participant characteristics

In this study, 8993 participants were included (Figure S1). Table 1 shows the characteristics of the participants according to their dietary caffeine intake. We grouped the participants according to their caffeine intake based on previous literature. Walter’s study defined 40–200 mg of caffeine per day as a "moderate" amount^29^. Further, Nawrot and van Dam RM research pointed out that more than 400 mg of caffeine daily is harmful to health^30,31^. Accordingly, we divided caffeine intake into four groups: ≥ 0 to < 40 mg/day, ≥ 40 to < 200 mg/day, ≥ 200 to < 400 mg/day, and ≥ 400 mg/day. Statistically significant differences were observed in age, educational level, race/ethnicity, marital status, PIR, BMI, smoking status, alcohol status, cancer, TG, energy intake, protein intake, calcium intake, magnesium intake, iron intake, and sodium intake in the different dietary caffeine intake groups (*P* < 0.05).

**Table 1:** Characteristics of study participants aged ≥ 20 years from 1999 to 2004 National Health and Nutrition Examination Survey.

Characteristics	Overall	Caffeine intake (mg/day)				*P* value
		Group 1	Group 2	Group 3	Group 4	
		(≥ 0 to < 40 mg/day)	(≥ 40 to < 200 mg/day)	(≥ 200 to < 400 mg/day)	(≥ 400 mg/day)	
Sample size, n (%)	8993 (100)	3038 (33.78)	3446 (38.32)	1644 (18.28)	865 (9.62)	
Age, y, mean (SD)	49.4 (18.8)	47.9 (20.0)	50.0 (19.4)	50.5 (17.00)	50.1 (14.8)	< 0.001
Sex, n (%)						< 0.001
Male	4234	1232 (40.55)	1586 (46.02)	876 (53.28)	540 (62.43)	
Female	4759	1806 (59.45)	1860 (53.98)	768 (46.72)	325 (37.57)	
Educational level, n (%)						< 0.001
< High school	3062 (34.12)	1191 (39.32)	1218 (35.42)	451 (27.45)	202 (23.41)	
Completed high school	2071 (23.08)	632 (20.86)	785 (22.83)	416 (25.32)	238 (27.58)	
> High school	3841 (42.80)	1206 (39.82)	1436 (41.76)	776 (47.23)	423 (49.02)	
Race/ethnicity, n (%)						< 0.001
Non-Hispanic White	4390 (48.82)	1085 (35.71)	1611 (46.75)	1030 (62.65)	664 (76.76)	
Non-Hispanic Black	1686 (18.75)	830 (27.32)	632 (18.34)	168 (10.22)	56 (6.47)	
Other	2917 (32.44)	1123 (36.97)	1203 (34.91)	446 (27.13)	145 (16.67)	
Marital status, n (%)						< 0.001
Married/living with partner	5351 (62.76)	1666 (57.41)	2054 (62.97)	1047 (67.42)	584 (72.19)	
Widowed/divorced/separated/never married	3175 (37.24)	1236 (42.59)	1208 (37.03)	506 (32.58)	225 (27.81)	
PIR, n (%)						< 0.001
< 1.00	1502 (18.59)	655 (24.34)	572 (18.49)	188 (12.47)	87 (11.04)	
1.00 to < 2.00	2098 (25.97)	731 (27.16)	867 (28.03)	336 (22.28)	164 (20.81)	
≥ 2.00	4480 (5545)	1305 (48.49)	1654 (53.48)	984 (65.25)	537 (68.15)	
BMI category, n (%)						0.024
< 25.0 kg/m^2^	2782 (31.97)	975 (33.31)	1018 (30.62)	521 (32.56)	268 (31.53)	
25.0 to < 30.0 kg/m^2^	3169 (36.42)	1071 (36.62)	1213 (36.48)	575 (35.94)	309 (36.35)	
≥ 30.0 kg/m^2^	2751 (31.61)	880 (30.06)	1094 (32.90)	504 (31.50)	273 (32.12)	
Smoking status, n (%)						< 0.001
Never smoking	4688 (52.20)	1913 (63.11)	1833 (53.27)	703 (42.79)	239 (27.63)	
Former smoker	2383 (26.54)	656 (21.64)	931 (27.06)	515 (31.35)	281 (32.49)	
Current smoker	1909 (21.26)	462 (15.24)	677 (19.67)	425 (25.87)	345 (39.88)	
Alcohol status, n (%)	5730 (66.95)	1659 (58.03)	2184 (66.81)	1203 (75.76)	684 (81.24)	< 0.001
Cancer, n (%)	756 (8.41)	218 (7.19)	321 (9.32)	149 (9.07)	68 (7.87)	0.013
Hypertension, n (%)	3961 (44.05)	1318 (43.38)	1586 (46.02)	711 (43.25)	346 (40.00)	0.007
TG, mmol/L, mean (SD)	5.20 (1.05)	5.15 (1.05)	5.21 (1.08)	5.25 (1.01)	5.23 (1.01)	0.011
Energy intake, kcal/day, mean (SD)	2084.33 (1014.68)	1941.82 (971.38)	2036.96 (982.85)	2242.28 (988.06)	2473.39 (1187.85)	< 0.001
Protein intake, g/day, mean (SD)	78.69 (42.92)	75.63 (43.52)	76.67 (41.54)	82.68 (41.83)	89.93 (45.84)	< 0.001
Calcium intake,mg/day, mean (SD)	803.14 (580.56)	788.32 (612.26)	785.70 (562.78)	826.30 (543.01)	880.64 (598.01)	< 0.001
Magnesium intake, mg/day, mean (SD)	274.17 (148.27)	265.21 (157.07)	260.07 (137.19)	287.91 (137.32)	335.68 (161.12)	< 0.001
Iron intake, mg/day, mean (SD)	15.03 (9.43)	14.75 (9.71)	14.60 (9.10)	15.52 (9.05)	16.79 (10.16)	< 0.001
Sodium intake, mg/day, mean (SD)	3231.35 (1815.33)	3013.37 (1759.74)	3125.67 (1707.05)	3510.66 (1861.69)	3887.05 (2100.39)	< 0.001

*SD* standard deviation, *PIR* poverty-income ratio (ratio of family income to poverty threshold), *BMI* body mass index (calculated as weight in kilograms divided by the square of height in meters), *TG* triglycerides.

Participants with the lowest dietary caffeine intake in group 1 (≥ 0 to < 40 mg/day) were likely to be younger, non-Hispanic white, less educated, living alone, poorer, and underweight, with less smoking, less drinking, no cancer, no hypertension, lower TG, and lower energy, protein, calcium, magnesium, iron, and sodium intake.

### Association between dietary caffeine intake and severe headache or migraine

We investigated the individual effects of each covariate on severe headaches or migraine using univariate analyses separately for the males and females in Table 2. In males, the incidence of severe headaches or migraines was likely to be with higher education, married/living with a partner, relatively wealthy, with greater BMI, drinking, hypertension, and cancer patients. Also, the higher incidence of severe headaches or migraines in females may be higher education, those who lived alone, were relatively wealthy, with less BMI, drinking, hypertension, and cancer patients.

**Table 2:** Effects of factors on severe headache or migraine in adults.

Variables	Males (n = 4234)			Females (n = 4759)		
	Statistics	OR (95% Cl)	*P-* value	Statistics	OR (95% Cl)	*P *value
Age	50.50 ± 18.49	0.98(0.98,0.99)	< 0.001	48.45 ± 19.05	0.98(0.97,0.98)	< 0.001
Educational level						
< High school	1496	Reference		1566	Reference	
Completed high school	957	0.81(0.64,1.01)	0.064	1114	0.79(0.66,0.94)	0.007
> High school	1772	0.62(0.51,0.76)	< 0.001	2069	0.75(0.65,0.87)	< 0.001
Race/ethnicity						
Non-Hispanic White	2108	Reference		2282	Reference	
Non-Hispanic Black	793	1.15(0.91,1.46)	0.231	893	1.46(1.22,1.74)	< 0.001
Other	1333	1.15(0.94,1.40)	0.168	1584	1.60(1.39,1.85)	< 0.001
Marital status						
Widowed/divorced/separated/never married	1254	Reference		1921	Reference	
Married/living with partner	2776	0.82(0.68,0.98)	0.032	2575	1.09(0.95,1.25)	0.209
PIR						
< 1.00	638	Reference		864	Reference	
1.00 to < 2.00	988	0.65(0.50,0.84)	< 0.001	1110	0.80(0.66,0.97)	0.021
≥ 2.00	2210	0.45(0.36,0.57)	< 0.001	2270	0.59(0.50,0.70)	< 0.001
BMI category						
< 25.0 kg/m^2^	1276	Reference		1506	Reference	
25.0 to < 30.0 kg/m^2^	1726	0.96(0.78,1.19)	0.723	1443	1.03(0.87,1.22)	0.709
≥ 30.0 kg/m^2^	1092	1.23(0.98,1.55)	0.071	1659	1.28(1.09,1.49)	0.002
Smoking status						
Never smoking	1730	Reference		2958	Reference	
Former smoker	1415	0.96(0.78,1.19)	0.734	968	0.84(0.71,1.00)	0.050
Current smoker	1083	1.60(1.30,1.97)	< 0.001	826	1.49(1.27,1.76)	< 0.001
Alcohol status						
No	750	Reference		2078	Reference	
Yes	3329	0.88(0.71,1.10)	0.264	2401	0.89(0.78,1.02)	0.089
Cancer						
No	3861	Reference		4368	Reference	
Yes	370	0.58(0.40,0.84)	0.004	386	0.93(0.73,1.18)	0.555
Hypertension						
No	2338	Reference		2694	Reference	
Yes	1896	0.94(0.78,1.12)	0.458	2065	0.73(0.64,0.83)	< 0.001
TG (mmol/L)	5.12 ± 1.08	0.94(0.86,1.02)	0.162	5.27 ± 1.08	0.88(0.82,0.94)	< 0.001
Energy intake (100 kcal/day)	24.10 ± 11.31	1.01(1.00,1.02)	0.007	17.94 ± 7.94	1.01(1.00,1.02)	0.004
Protein intake (100 g/day)	0.92 ± 0.48	1.20(1.01,1.42)	0.039	0.67 ± 0.34	1.09(0.90,1.31)	0.381
Calcium intake (100 mg/day)	8.82 ± 6.40	1.01(0.99,1.02)	0.283	7.33 ± 5.12	1.00(0.99,1.01)	0.754
Magnesium intake (100 mg/day)	3.13 ± 1.66	1.01(0.95,1.06)	0.821	2.40 ± 1.21	0.95(0.89,1.00)	0.044
Iron intake (100 mg/day)	0.17 ± 0.10	1.31(0.58,2.98)	0.520	0.13 ± 0.08	0.50(0.22,1.13)	0.095
Sodium intake (100 mg/day)	37.13 ± 20.30	1.00(1.00,1.01)	0.271	28.03 ± 14.73	1.00(1.00,1.01)	0.347

*SD* standard deviation, *PIR* poverty-income ratio, *BMI* body mass index, *TG* triglyceride, *CI* confidence interval, *OR* odds ratio.

The fully adjusted model observed a linear relationship between dietary caffeine intake and severe headaches or migraines in US adults (males and females) (Figure S2). A scatter plot of dietary caffeine intake and severe headaches or migraine is shown in Figure S3.

The results of the multivariate logistic regression analysis are shown in Table 3. After adjusting for confounders, a significant association between dietary caffeine intake and severe headaches or migraines was detected in Models 1–4. In Model 4, all variables were adjusted; for every 100 mg/day increase in dietary caffeine intake, severe headache or migraine incidence increased by 5% (OR 1.05, 95% CI 1.02–1.07, *P* < 0.001) in all adults. Notably, for every 100 mg/day increase in dietary caffeine intake, severe headache or migraine incidence increased by 5% (OR 1.05, 95% CI 1.01–1.08, *P* = 0.006) and 7% (OR 1.07, 95% CI 1.02–1.11, *P* = 0.002) in males and females, respectively.

**Table 3:** Associations of caffeine intake with severe headache or migraine.

Variables	Crude model		Model 1^a^		Model 2^b^		Model 3^c^		Model 4^d^	
	OR (95% CI)	*P* value	OR (95% CI)	*P* value	OR (95% CI)	*P* value	OR (95% CI)	*P* value	OR (95% CI)	*P* value
Totals										
Per 100 mg/day caffeine intake	1.01 (0.98,1.03)	0.546	1.03 (1.01,1.05)	0.016	1.03 (1.01,1.06)	0.006	1.04 (1.01,1.06)	0.003	1.05 (1.02,1.07)	< 0.001
Group 1 (Caffeine:(≥ 0 to < 40 mg/day)	Reference^1^		Reference^1^		Reference^1^		Reference^1^		Reference^1^	
Group 2 (Caffeine: ≥ 40 to < 200 mg/day)	1.00 (0.89,1.13)	0.979	1.08 (0.95,1.22)	0.223	1.11 (0.98,1.26)	0.110	1.10 (0.97,1.25)	0.123	1.12 (0.98,1.27)	0.090
Group 3 (≥ 200**t**o < 400 mg/day)	0.95 (0.82,1.10)	0.497	1.09 (0.93,1.28)	0.268	1.18 (1.00,1.38)	0.048	1.18 (1.01,1.39)	0.040	1.24 (1.05,1.46)	0.011
Group 4 (≥ 400 mg/day)	1.02 (0.85,1.23)	0.838	1.23 (1.01,1.49)	0.040	1.28 (1.05,1.57)	0.016	1.30 (1.06,1.60)	0.011	1.42 (1.16,1.75)	< 0.001
*P* value for trend	0.857		0.047		0.005		0.003		< 0.001	
Males										
Per 100 mg/day caffeine intake	1.04 (1.01,1.07)	0.019	1.04 (1.01,1.07)	0.008	1.04 (1.01,1.07)	0.012	1.04 (1.01,1.08)	0.007	1.05 (1.01,1.08)	0.006
Group 1 (Caffeine:(≥ 0 to < 40 mg/day)	Reference^1^		Reference^1^		Reference^1^		Reference^1^		Reference^1^	
Group 2 (Caffeine: ≥ 40 to < 200 mg/day)	1.07 (0.86,1.33)	0.548	1.10 (0.88,1.38)	0.399	1.11 (0.88,1.39)	0.380	1.12 (0.89,1.40)	0.342	1.11 (0.88,1.40)	0.378
Group 3 (≥ 200 to < 400 mg/day)	1.08 (0.84,1.39)	0.545	1.15 (0.89,1.50)	0.286	1.22 (0.93,1.59)	0.152	1.23 (0.94,1.61)	0.126	1.23 (0.94,1.62)	0.131
Group 4 (≥ 400 mg/day)	1.37 (1.03,1.81)	0.028	1.48 (1.11,1.99)	0.009	1.51 (1.11,2.06)	0.009	1.54 (1.13,2.10)	0.006	1.56 (1.14,2.14)	0.005
*P* value for trend	0.033		0.007		0.004		0.002		0.002	
Females										
Per 100 mg/day caffeine intake	1.02 (0.99,1.06)	0.184	1.07 (1.03,1.11)	< 0.001	1.06 (1.02,1.10)	0.005	1.06 (1.02,1.10)	0.003	1.07 (1.02,1.11)	0.002
Group 1 (Caffeine: ≥ 0 to < 40 mg/day)	Reference^1^		Reference^1^		Reference^1^		Reference^1^		Reference^1^	
Group 2 (Caffeine: ≥ 40 to < 200 mg/day)	1.04 (0.89,1.20)	0.630	1.17 (1.00,1.36)	0.045	1.17 (1.00,1.36)	0.048	1.16 (0.99,1.36)	0.059	1.15 (0.99,1.35)	0.071
Group 3 (≥ 200**t**o < 400 mg/day)	1.04 (0.86,1.26)	0.710	1.28 (1.05,1.56)	0.016	1.28 (1.04,1.58)	0.018	1.29 (1.05,1.59)	0.017	1.30 (1.05,1.60)	0.015
Group 4 (≥ 400 mg/day)	1.10 (0.84,1.43)	0.489	1.43 (1.08,1.88)	0.011	1.34 (1.00,1.79)	0.046	1.36 (1.02,1.81)	0.038	1.40 (1.04,1.87)	0.026
*P* value for trend	0.593		0.003		0.007		0.007		0.006	

*CI* confidence interval, *OR* odds ratio.

^a^Model 1: Adjust for age and race/ethnicity.

^b^Model 2: Adjusted for the variables in Model 1 plus body mass index, poverty-income ratio, educational level, marital status, alcohol status, and smoking status.

^c^Model 3: Adjusted for the variables in Model 2 plus hypertension, cancer, and triglycerides.

^d^Model 4: Adjusted for the variables in Model 3 plus energy intake, protein intake, calcium intake, magnesium intake, iron intake, and sodium intake.

Among the groups of dietary caffeine intake in Model 4 compared with participants in the first group of dietary caffeine intake (≥ 0 to < 40 mg/day), while the fourth group of dietary caffeine intake (≥ 400 mg/day) was associated with severe headaches or migraine (OR 1.42, 95% CI 1.16–1.75, *P* < 0.001). These relationships are also found in males and females.

The results of subgroup analyses by age, educational level, race/ethnicity, marital status, BMI, cancer, and energy intake are presented in Table S2. A significant interaction of dietary caffeine intake with age was found (*P* < 0.05).

## Discussion

The results of this cross-sectional study of adult males in the US showed the association between dietary caffeine intake and severe headaches or migraine for the first time in a nationally representative sample. After adjusting for other variables, we found a linear relationship between dietary caffeine intake and severe headaches or migraines from 1999 to 2004. We also found that age modified the association.

The study investigated increased odds of severe headaches or migraine with increasing dietary caffeine intake in adults.

Our data were obtained from the NHANES database, which surveys a nationally representative sample of various health and nutrition measures covering diverse demographic characteristics such as multi-ethnicity^28^. Furthermore, the database is comprehensive, and its results are representative.

Caffeine is structurally similar to adenosine^12^, which plays important roles in regulating neurotransmitter release in the brain, movement, reward, sleep/wakefulness, cognition, and analgesia^32^. When caffeine binds to adenosine receptors on the cell surface, it acts as an adenosine receptor antagonist, which in turn induces cortical hyperexcitability and maintains arousal in the brain^11,13^. The main effect of caffeine in the neuroendocrine control system is the activation of distinct neuronal pathways by altering neurotransmitter release, which causes headaches and dependence^33^. Dehydration is also considered a possible contributor to migraine, and higher doses of caffeine can induce acute diuresis, which may subsequently lead to dehydration^34,35^. Chronic caffeine intake promotes a nociceptive state of cortical hyperexcitability and excitable neurons by antagonizing G protein-coupled purinergic (P1) receptors, which induces or exacerbates headaches^14,36^. In addition, chronic repetitive exposure to caffeine increases the risk of developing analgesic-overuse headaches, chronic daily headaches, and physical dependency^37–39^.

Age may affect the relationship between dietary caffeine intake and severe headaches or migraine; in the subgroup analysis, we found a stronger relationship in age < 60 years, and the interaction is significant among females. Hormones, such as menarche, oral contraceptive use, pregnancy, menopause, etc., greatly influence females’ migraines^40^, mainly involving young and middle-aged people ≥ 20 to < 60 years. Studies also have shown that caffeine intake affects the levels of luteal progesterone levels, luteal total, and free estradiol in premenopausal women; in postmenopausal females, no significant associations were detected with these hormones^41^, which may have implications for the relationship between dietary caffeine intake and severe headaches or migraine in adults non-elderly. However, its impact mechanism is still unclear, and it is necessary to conduct further studies to explore this conclusively.

This study had some limitations. First, this was a cross-sectional study; thus, we could not determine a causal relationship between dietary caffeine intake and severe headaches or migraine. Second, the data were obtained from questionnaires; therefore, there could be significant recall bias. Third, severe headache or migraine is based on self-reports and cannot be distinguished by type. Fourth, the caffeine intake calculated by food conversion may be inaccurate. However, the data used in this study came from the NHANES database, a research program designed to assess the health and nutritional status of adults and children in the US, and is intended to be accurate^42^. Fifth, the results may have been influenced by uncontrolled confounding, such as non-alcoholic fatty liver disease (NAFLD)^43^. Sixth, the data is nearly 20 years old at this point and may not be reflective of the current population, so our next study will include more recent data.

## Conclusions

Our study showed that higher dietary caffeine intake is positively associated with a higher prevalence of severe headaches or migraines in US adults. However, further prospective studies are needed to clarify whether increased dietary caffeine intake increases the risk of severe headaches or migraine.

### Supplementary Information

Supplementary Information.

## Data Availability

The datasets used and analyzed during the current study are available from the corresponding author upon reasonable request.
