# Publisher Correction: Association between dietary caffeine intake and severe headache or migraine in US adults

**DOI:** 10.1038/s41598-023-43648-z

**Published:** 2023-09-29

**Authors:** Lu Zhang, Jiahui Yin, Jinling Li, Haiyang Sun, Yuanxiang Liu, Jiguo Yang

**Affiliations:** 1 The First Clinical College, Shandong University of Traditional Chinese Medicine, Jinan, China; 2 College of Traditional Chinese Medicine, Shandong University of Traditional Chinese Medicine, Jinan, China; 3 College of Acupuncture and Massage, Shandong University of Traditional Chinese Medicine, Jinan, China; 4 The Second Clinical Medical College, Guangzhou University of Chinese Medicine, Guangzhou, China; 5 Department of Neurology, Affiliated Hospital of Shandong University of Traditional Chinese Medicine, Jinan, China

Correction to: *Scientific Reports* 10.1038/s41598-023-36325-8, published on 23 June 2023

In the original version of this Article Jinling Li was incorrectly affiliated with ‘The Second Clinical Medical College, Guangzhou University of Chinese Medicine, Guangzhou, China’. The correct affiliation is listed below.

College of Acupuncture and Massage, Shandong University of Traditional Chinese Medicine, Jinan, China

Also, Haiyang Sun was incorrectly affiliated with “College of Acupuncture and Massage, Shandong University of Traditional Chinese Medicine, Jinan, China.” The correct affiliation is listed below

The Second Clinical Medical College, Guangzhou University of Chinese Medicine, Guangzhou, China

The original Article has been corrected.

