# Supplementary material for: Association between dietary caffeine intake and severe headache or migraine in US adults
Source: Sci Rep. 2023 Jun 23;13:10220. doi: 10.1038/s41598-023-36325-8 (PMC10290098; doi:10.1038/s41598-023-36325-8)
Supplement: Supplementary file 1 — Supplementary Information. [file 41598_2023_36325_MOESM1_ESM.docx]

**Association Between Dietary Caffeine Intake and Severe**

**Headache or Migraine in US Adults**

**Lu Zhang^1,†,^ Jiahui Yin^2,†^ , Jinling Li^3,†^ , Haiyang Sun^4^ , Yuanxiang Liu^5,*^ , Jiguo Yang^3,*^**

^1^The First Clinical College, Shandong University of Traditional Chinese Medicine,

Jinan, China

^2^College of Traditional Chinese Medicine, Shandong University of Traditional

Chinese Medicine, Jinan, China

^3^College of Acupuncture and Massage, Shandong University of Traditional Chinese

Medicine, Jinan, China

^4^The Second Clinical Medical College of Guangzhou University of Chinese Medicine,

Guangzhou, China

^5^Department of Neurology, Affiliated Hospital of Shandong University of Traditional

Chinese Medicine, Jinan, China

†These authors contributed equally to this work.

**Correspondence:**

Yuanxiang Liu

lyxlwtg@126.com

**Table S1.** Missing covariates.

| Variable | Number of patients (% missing) |
| --- | --- |
| Age | 0 (0%) |
| Educational level | 19 (0.21%) |
| Race/ethnicity | 0 (0%) |
| Marital status | 467 (5.19%) |
| PIR | 913 (10.15%) |
| BMI category | 291 (3.24%) |
| Smoking status | 13 (0.14%) |
| Alcohol status | 435 (4.84%) |
| Cancer | 8 (0.09%) |
| Hypertension | 0 (0%) |
| TG | 536 (5.96%) |
| Energy intake | 0 (0%) |
| Protein intake | 0 (0%) |
| Calcium intake | 0 (0%) |
| Magnesium intake | 0 (0%) |
| Iron intake | 0 (0%) |
| Sodium intake | 0 (0%) |

Abbreviations: PIR, poverty-income ratio; BMI, body mass index; TG, triglycerides.

**Table S2.** Subgroup analysis of the effect of caffeine intake on severe headache or migraine.

| Subgroup | Totals | | | Males | | | Females | | |
| --- | --- | --- | --- | --- | --- | --- | --- | --- | --- |
|  | Number of participants | OR (95%CI) | *P* for interaction | Number of participants | OR (95%CI) | *P* for interaction | Number of participants | OR (95% CI) | *P* for interaction |
| Overall | 8993 | 1.05 (1.02,1.07) |  | 4234 | 1.05 (1.01,1.08) |  | 4759 | 1.07 (1.02,1.11) |  |
| Age (years) |  |  | 0.013 |  |  | 0.142 |  |  | 0.014 |
| <60 | 5924 | 1.04 (1.02,1.07) |  | 2718 | 1.05 (1.01,1.08) |  | 3206 | 1.05 (1.01,1.10) |  |
| ≥60 | 3069 | 0.95 (0.89,1.02) |  | 1516 | 0.98 (0.90,1.07) |  | 1553 | 0.92 (0.82,1.03) |  |
| Education |  |  | 0.588 |  |  | 0.591 |  |  | 0.361 |
| <High school | 3062 | 1.04 (1.00, 1.09) |  | 1496 | 1.04(0.99,1.09) |  | 1566 | 1.04 (0.97, 1.12) |  |
| Completed high school | 2071 | 1.07 (1.02, 1.11) |  | 957 | 1.03 (0.96,1.09) |  | 1114 | 1.12 (1.05,1.19) |  |
| >High school | 3841 | 1.04 (1.00, 1.08) |  | 1772 | 1.06 (1.01,1.12) |  | 2069 | 1.04 (0.98,1.11) |  |
| Race/ethnicity |  |  | 0.960 |  |  | 0.753 |  |  | 0.851 |
| Non-Hispanic white | 4390 | 1.05 (1.02,1.08) |  | 2108 | 1.04 (1.00,1.08) |  | 2282 | 1.06 (1.01,1.11) |  |
| Non-Hispanic black | 1686 | 1.06 (0.99, 1.13) |  | 793 | 1.08 (0.99,1.17) |  | 893 | 1.05 (0.91,1.21) |  |
| Other | 2917 | 1.05 (0.99, 1.11) |  | 1333 | 1.04 (0.96,1.13) |  | 1584 | 1.09 (1.00,1.18) |  |
| Marital status |  |  | 0.886 |  |  | 0.760 |  |  | 0.457 |
| Married/Living with a partner | 5351 | 1.04 (1.01, 1.08) |  | 2776 | 1.03 (1.00,1.07) |  | 2575 | 1.08 (1.03,1.14) |  |
| Widowed/Divorced/Separated/Never married | 3175 | 1.05 (1.01, 1.10) |  | 1254 | 1.04 (0.99,1.10) |  | 1921 | 1.03 (0.97, 1.11) |  |
| BMI category |  |  | 0.971 |  |  | 0.911 |  |  | 0.762 |
| <25.0 kg/m2 | 2782 | 1.05 (1.01,1.08) |  | 1276 | 1.04 (0.99,1.08) |  | 1506 | 1.08 (1.02,1.15) |  |
| 25.0 to <30.0 kg/m2 | 3169 | 1.05 (1.00,1.10) |  | 1726 | 1.06 (1.00,1.12) |  | 1443 | 1.06 (0.99,1.14) |  |
| ≥30.0 kg/m2 | 2751 | 1.05 (1.00,1.09) |  | 1092 | 1.06 (0.99,1.12) |  | 1659 | 1.05 (0.98,1.12) |  |
| Cancer |  |  | 0.952 |  |  | 0.988 |  |  | 0.952 |
| No | 8229 | 1.05 (1.02,1.07) |  | 3861 | 1.05 (1.01,1.08) |  | 4368 | 1.06 (1.02,1.11) |  |
| Yes | 756 | 1.06 (0.97,1.17) |  | 370 | 1.06 (0.91,1.23) |  | 386 | 1.09 (0.96,1.23) |  |
| Hypertension |  |  | 0.892 |  |  | 0.299 |  |  | 0.100 |
| No | 5032 | 1.05 (1.02,1.08) |  | 2338 | 1.03 (1.00,1.07) |  | 2694 | 1.09 (1.04,1.15) |  |
| Yes | 3961 | 1.05 (1.01,1.09) |  | 1896 | 1.07 (1.01,1.13) |  | 2065 | 1.02 (0.96,1.09) |  |
| Energy intake |  |  | 0.336 |  |  | 0.312 |  |  | 0.202 |
| Low | 2998 | 1.07 (1.01,1.13) |  | 1411 | 1.11 (1.02,1.21) |  | 1586 | 1.03 (0.95,1.10) |  |
| Middle | 2995 | 1.07 (1.02,1.12) |  | 1411 | 1.03 (0.98,1.09) |  | 1585 | 1.11 (1.04,1.19) |  |
| High | 3000 | 1.03 (1.00,1.07) |  | 1412 | 1.04 (1.00,1.09) |  | 1588 | 1.05 (0.99,1.12) |  |

Abbreviations: PIR, poverty-income ratio; BMI, body mass index; TG, triglycerides.

Analyses were adjusted for all covariates. Stratified variables were not adjusted in the corresponding models.


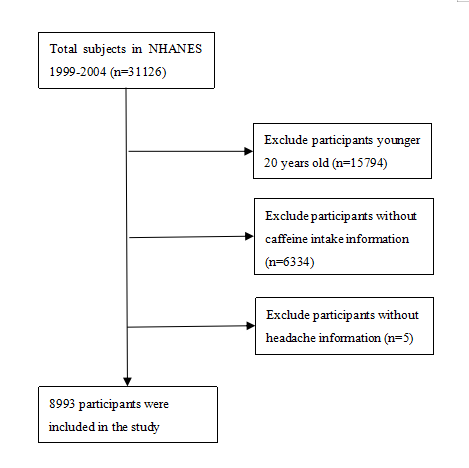


**Figure S1.** Flowchart for inclusion of study participants

**
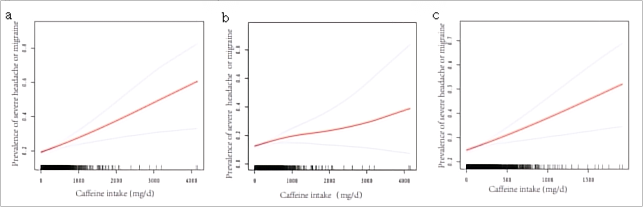
**

**Figure S2.** Association between dietary caffeine intake and severe headache or migraine in US adults (n=8993), which was adjusted for age, race/ethnicity, body mass index, poverty-income ratio, educational level, marital status, hypertension, cancer, energy intake, protein intake, calcium intake, magnesium intake, iron intake, sodium intake, alcohol status, smoking status, and triglycerides. The black vertical line on the horizontal axis represents the caffeine distribution, the red line represents the best fit, and the difference between the dashed lines represents the 95% confidence interval. (**a**) was in US adults. (**b**) was in US adult males. (**c**)was in US adult females.


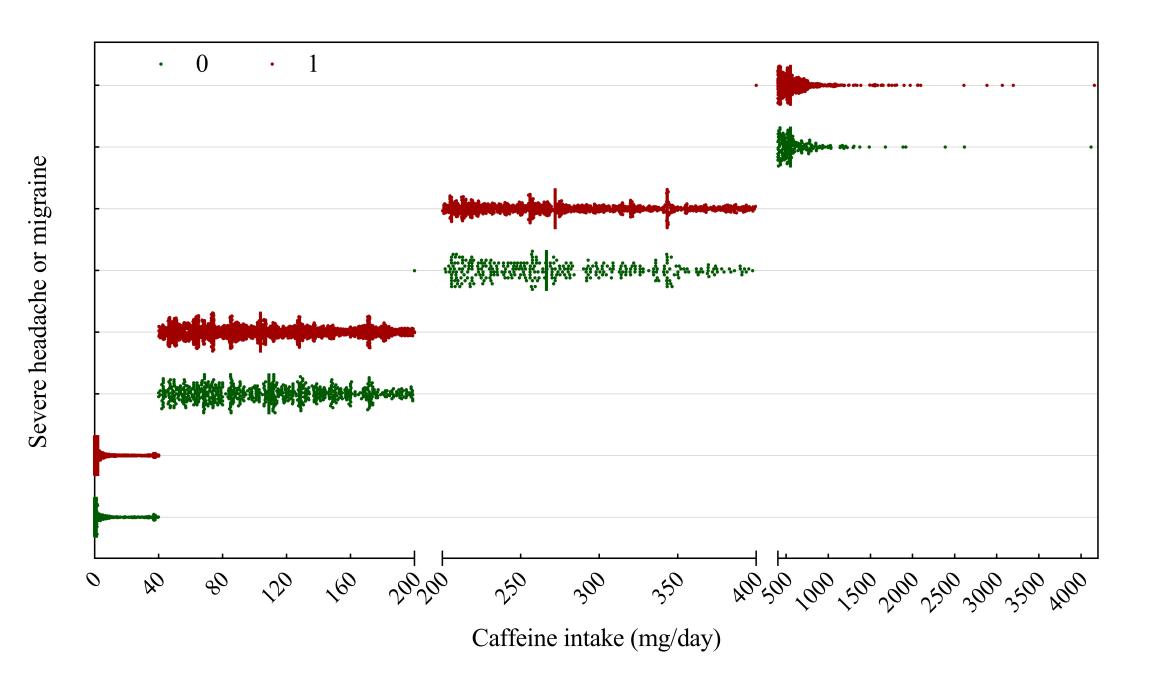


**Figure S3.** Association between dietary caffeine intake and severe headache or migraine scatter plot in US adults (n=8993). Green dots indicate no severe headache or migraine, and red dots indicate severe headache or migraine.
